# Multi-institution model (big model) versus single-institution model of knowledge-based volumetric modulated arc therapy (VMAT) planning for prostate cancer

**DOI:** 10.1038/s41598-022-19498-6

**Published:** 2022-09-10

**Authors:** Jun-ichi Fukunaga, Mikoto Tamura, Yoshihiro Ueda, Tatsuya Kamima, Yumiko Shimizu, Yuta Muraki, Kiyoshi Nakamatsu, Hajime Monzen

**Affiliations:** 1 Division of Radiology, Department of Medical Technology, Kyushu University Hospital, 3-1-1, Maidashi, Higashi-ku, Fukuoka, 812-8582 Japan; 2 Department of Medical Physics, Graduate School of Medical Sciences, Kindai University, 377-2, Ohno-Higashi, Osakasayama, Osaka 589-8511 Japan; 3 Department of Radiation Oncology, Osaka International Cancer Institute, 3-1-69, Otemae, Chuo-ku, Osaka, 537-8567 Japan; 4 Radiation Oncology Department, Cancer Institute Hospital, Japanese Foundation for Cancer Research, 3-8-31 Ariake, Koto-ku, Tokyo, 1358550 Japan; 5 Department of Radiology, Seirei Hamamatsu General Hospital, 2-12-12 Sumiyoshi, Naka Ward,, Hamamatsu, Shizuoka 430-8558 Japan; 6 Department of Radiation Oncology, Faculty of Medicine, Kindai University, 377-2, Ohno-Higashi, Osakasayama, Osaka, 589-8511 Japan

**Keywords:** Cancer, Oncology, Physics

## Abstract

We established a multi-institution model (big model) of knowledge-based treatment planning with over 500 treatment plans from five institutions in volumetric modulated arc therapy (VMAT) for prostate cancer. This study aimed to clarify the efficacy of using a large number of registered treatment plans for sharing the big model. The big model was created with 561 clinically approved VMAT plans for prostate cancer from five institutions (A: 150, B: 153, C: 49, D: 60, and E: 149) with different planning strategies. The dosimetric parameters of planning target volume (PTV), rectum, and bladder for two validation VMAT plans generated with the big model were compared with those from each institutional model (single-institution model). The goodness-of-fit of regression lines (R^2^ and χ^2^ values) and ratios of the outliers of Cook’s distance (CD) > 4.0, modified Z-score (mZ) > 3.5, studentized residual (SR) > 3.0, and areal difference of estimate (dA) > 3.0 for regression scatter plots in the big model and single-institution model were also evaluated. The mean ± standard deviation (SD) of dosimetric parameters were as follows (big model vs. single-institution model): 79.0 ± 1.6 vs. 78.7 ± 0.5 (D_50_) and 0.13 ± 0.06 vs. 0.13 ± 0.07 (Homogeneity Index) for the PTV; 6.6 ± 4.0 vs. 8.4 ± 3.6 (V_90_) and 32.4 ± 3.8 vs. 46.6 ± 15.4 (V_50_) for the rectum; and 13.8 ± 1.8 vs. 13.3 ± 4.3 (V_90_) and 39.9 ± 2.0 vs. 38.4 ± 5.2 (V_50_) for the bladder. The R^2^ values in the big model were 0.251 and 0.755 for rectum and bladder, respectively, which were comparable to those from each institution model. The respective χ^2^ values in the big model were 1.009 and 1.002, which were closer to 1.0 than those from each institution model. The ratios of the outliers in the big model were also comparable to those from each institution model. The big model could generate a comparable VMAT plan quality compared with each single-institution model and therefore could possibly be shared with other institutions.

## Introduction

Intensity-modulated radiotherapy (IMRT) and volumetric modulated arc therapy (VMAT) treatment planning require trial and error during the optimization process to obtain an ideal dose distribution. The plan quality for IMRT and VMAT depends on the knowledge and experience of the planner or institution during optimization, which can cause large intra- and inter-institutional variability^1–5^, and sometimes even affect treatment outcomes^6^.

RapidPlan (RP) (Varian Medical Systems, Palo Alto, CA, USA), a knowledge-based planning software, uses a model library containing the dose-volume histogram (DVH) of previous treatment plans. It automatically provides optimization objectives for future patients based on a trained model for VMAT planning. Previous studies concluded that RP with a single optimization could create clinically acceptable VMAT plans for prostate cancer, and could also reduce the optimization time independently of the planner’s skill level and knowledge^7^. Furthermore, it was expected that RP would be shared among institutions and thereby standardize the plan quality between them^8–11^. However, sharing the single-institution model with multiple institutions remained a challenge, because RP depended on registered plans, including the planning strategies at each institution, such as the prescribing method to the targets and the dose constraint of the organs at risk (OARs)^12^.

Panettieri et al. attempted to share a model trained with 110 treatment plans from multiple institutions that had different irradiation methods (IMRT and VMAT), contouring, planning strategies, and prescription doses contributing to reducing the intra- and inter-institutional variability^13^. However, all the plans in the multi-institution model were standardized by achieving the DVH constraints of their group. Therefore, the sharing of their multi-institution model was limited to the institutions that had the different planning strategies and experience.

We hypothesized that the model with a large number of plans could be applied to the various planning strategies. To examine this, we established and evaluated a multi-institution model (big model) that aggregated over 500 treatment plans from five institutions with different planning strategies and constraints for targets and OARs in prostate cancer VMAT. In this study, we compared the big model with each institutional model (single-institution model) by using the dosimetric parameters of the planning target volume (PTV), rectum, and bladder for two validation VMAT plans. The efficacy of the big model, including the large number of registered treatment plans, and the potential to reduce the inter-variability of the plan quality were clarified to be able to share it.

## Methods

### Institutions and plan design

Five institutions (A–E) that treated prostate cancer cases with VMAT in Japan were enrolled. The definition of gross tumor volume, the margins defining the clinical target volume (CTV) and PTV in each direction, and the dose constraints have been described in a previous study^12,14^. Table 1 shows the dose constraints used by each institution. The five institutions had different planning strategies. All methods were performed in accordance with the relevant guideline.

**Table 1:** Dose constraints at each institution.

Institution	OARs			Target
A	Rectal wall	Bladder wall	CTV	PTV
	V_78_ ≤ 0.1 cc	V_70_ ≤ 35%	D_min_ ≥ 100%	D_50_ = 100%
	V_70_ ≤ 25%	V_40_ ≤ 60%		
	V_60_ ≤ 35%			
	V_40_ ≤ 60%			
B	Rectal wall	Bladder wall	CTV	PTV sub. Rectum
	V_78_ < 1%	V_70_ < 35%		D_mean_ < 103%
	V_70_ < 20%	V_40_ < 60%		D_min_ > 99%
	V_60_ < 30%			D_max_ < 110%
	V_40_ < 60%			D_95_ = 100%
C	Rectum	Bladder	CTV	PTV
	V_70_ ≤ 5%	V_80_ ≤ 5%	D_98_ ≥ 98%	D_mean_ = 100%
	V_65_ ≤ 10%	V_75_ ≤ 15%	D_2_ ≤ 105%	D_95_ ≥ 95%
	V_60_ ≤ 20%	V_70_ ≤ 25%		V_90_ ≥ 98%
	V_40_ ≤ 40%	V_60_ ≤ 40%		D_2_ ≤ 105%
D	Rectum sub. PTV	Bladder sub. PTV	CTV	PTV sub. (rectum and bladder)
	D_50_ ≤ 69.7%	D_5_ ≤ 78.9%	D_95_ = 100%	68.4% ≤ D_5_ ≤ 71.1%
	D_5_ ≤ 78.9%	D_50_ ≤ 72.4%	88.2% ≤ D_5_ ≤ 92.1%	65.8% ≤ D_50_ ≤ 71.1%
			85.5% ≤ D50 ≤ 88.2	64.5% ≤ D95 ≤ 68.4%
			81.6% ≤ D95 ≤ 85.5	
E	Rectal wall	Bladder wall	CTV	PTV
	V_78_ < 1%	V_70_ < 35%		D_mean_ = 100%
	V_70_ < 20%	V_40_ < 60%		D_95_ ≥ 95%
	V_60_ ≤ 35%			V_90_ ≥ 98%
	V_40_ < 60%			D_max_ ≤ 110%

*CTV* Clinical target volume; *PTV* Planning target volume; *D*_*mean*_ Mean dose; *D*_*min*_ Minimum dose; *D*_*max*_ Maximum dose; *V*_*80*_*, V*_*78*_*, V*_*70*_*, V*_*65*_*, V*_*60*_*, and V*_*40*_, Organ at risk (OAR) volume ratio receiving doses exceeding 80 Gy, 78 Gy, 70 Gy, 65 Gy, 60 Gy, and 40 Gy, respectively; *V*_*90*_, Volume ratio receiving 90% of the prescribed dose; *D*_*95*_*, D*_*50*_*, D*_*5*_*, and D*_*2*_, Dose received by at least 95%, 50%, 5%, and 2% of the volume, respectively.

### Development of the single-institution model and the big model

An RP model is a mathematical model that uses knowledge from the included treatment plans to generate the estimated DVH and estimate-based objectives in the optimization process. The RP algorithm was explained in detail by Fogliata et al.^15^. The single-institution model and big model for RP were created using the prostate VMAT plans for clinical use at each institution. The number of single-institution models of registered cases in institutions A, B, C, D, and E were 123, 53, 20, 60, and 100, respectively. To build the big model, 561 approved clinical plans, including 150 from A, 153 from B, 49 from C, 60 from D, and 149 from E, were anonymized and submitted by each institution. These clinical plans were used at each institution from April 2017 to April 2019. The clinical plans used to configure the single-institution model were also registered in the big model, and the outliers were not excluded.

### Validation of each model

Two sets of computed tomography (CT) data and structures (cases I and II) used at institution B were anonymized and delivered to other institutions. CT image thickness was 2.5 mm and the field of view was 50 cm. The target and OARs were contoured by a radiation oncologist according to the protocol of institution B. The bladder volume was 83.8 cm^3^ in case I and 181.8 cm^3^ in case II. The planners who had sufficient experience with using RapidPlan at each institution calculated the dose distributions with the single-institution model and big model using Eclipse ver. 13.0 or 15.6 (Varian). The objective settings for the big model, as shown in Table 2, were the same as the settings of the single-institution model of each institution.

**Table 2:** Objective settings for optimization with the big model and each single-institution model.

Institution	Organ	Objective	Volume	Dose	gEUD	Priority		Objective	Volume	Dose	Priority
A	Rectum	Upper	0%	100%		Generate	CTV	Upper	0%	104%	Generate
		Line				Generate		Lower	100%	100%	Generate
	Bladder	Upper	0%	100%		Generate	PTV	Upper	0%	104%	Generate
		Line				Generate		Lower	100%	90%	Generate
B	Rectum wall	Line				Generate	PTV	Upper	0%	102%	120
	Bladder wall	Line				Generated		Lower	100%	99%	120
C	Rectum	Upper	0%	100%		0	PTV	Upper	0%	100%	115
		Upper gEUD		69.2%	9	Generate		Lower	100%	93.0%	Generate
		Line				Generate		Lower	97%	96.0%	Generate
	Bladder	Line				Generate		Lower	95.50%	98.0%	Generate
								Lower	90%	100.0%	Generate
D	Rectum	Upper	0%	76.9%		85	CTV	Upper	0%	100.0%	Generate
		Line				Generate		Lower	95%	97%	Generate
	Bladder	Upper	0%	79.5%		75		Lower	100%	94.9%	Generate
		Line				Generate	PTV	Upper	0%	94.9%	Generate
E	Rectum	Upper	0%	99%		Generate	CTV	Upper	0%	102.6%	Generate
		Upper gEUD		76.9%	10	Generate		Lower	100%	99%	Generate
		Line				Generate	PTV	Upper	0%	103%	Generate
	Bladder	Line				Generate		Lower	100%	90%	Generate
								Lower	96%	96%	Generate

*gEUD* Generalized equivalent uniform dose; *CTV* Clinical target volume; *PTV* Planning target volume.

To evaluate the dose distributions calculated with the single-institution model and big model, the minimum dose (in Gy) to 2%, 50%, 95%, and 98% (D_2_, D_50_, D_95_, and D_98_) of the PTV and the volume ratio receiving 90%, 80%, and 50% of the prescribed dose (V_90_, V_80_, and V_50_) for the rectum and bladder were calculated in two cases. The homogeneity index (HI; defined as HI = [D_2_–D_98_]/D_50_) was calculated. In this study, a dose prescription of 78 Gy (in 39 fractions) was used for the calculation. The differences of dosimetric parameters between the single-institution model (D_s_) and big model (D_b_) were calculated as follows:

$${\text{Difference }} = {\text{ D}}_{{\text{s}}} - {\text{ D}}_{{\text{b}}}$$

### Model analysis

In RP, the principal component analysis between geometrical dose-volume histogram (GEDVH) and actual DVH was performed. The regression model with the principal component score (PCS) of GEDVH and DVH was used to estimate the ideal DVH for a new case, which indicated the performance of its estimation. The goodness-of-fit for the regression models, coefficient of determination (R^2^), and average chi squared (χ^2^) value were evaluated. The R^2^ value ranges from 0 to 1, with a larger value indicating a better model fit. The χ^2^ value closer to 1.0 provides more certainty that the quality of the regression model is good. In addition, to evaluate the outliers of the rectum and bladder in each model, the following four parameters were evaluated: Cook’s distance (CD), modified Z-score (mZ), studentized residual (SR), and areal difference of estimate (dA). CD indicates the influential data points in a regression model. A high CD value has a significant effect on the regression line. The mZ value measures the difference of an individual geometric parameter from the median value in a training set and identifies geometric outliers. The SR value measures the difference of PCSs of the DVHs between the original data and the estimated data (e.g., first PCS of the original DVH versus first PCS of the estimated DVH), which reveals dosimetric outliers. The dA value indicates the difference between the estimated dose distribution and the actual one, and is essentially the difference between the estimated DVH curve and the actual DVH curve.

To investigate whether each institution model’s and big model’s training sets covered the geometrical characteristics of cases I and II, such as targets, rectum, and bladder, we investigated whether the following parameters were within the threshold of two standard deviations from the median of the training set: target and OAR volumes, OAR out-of-field volume percentage, OAR overlap volume percentage to target, and geometric distribution PCS. A more detailed description of the RP and DVH estimation algorithm can be found in reference^16^.

### Statistical analysis

For analysis among plans under the single-institution model and big model, the paired Wilcoxon signed-rank test was performed to calculate the differences in dosimetric parameters using JMP Pro 16 software (SAS Institute, Inc., Cary, NC, USA). A *P*-value < 0.05 was considered significant.

### Ethics approval and consent to participate

This study was approved at all Institutional Ethical Review Committees. (Kindai University Review Board No. 31–273, Kyushu University Review Board No. 2020–286, JFCR Review Board No. 2020–1049, Seirei Hamamatsu General Hospital Review Board No. 3333, Osaka International Cancer Institute Review Board No. 20050).

## Results

### Dosimetric parameters for the PTV, rectum, and bladder

Table 3 shows the mean and standard deviation (SD) values of dosimetric parameters for the PTV, rectum, and bladder that were calculated with each single-institution model and big model. There were no significant differences in the dosimetric parameters (*P* > 0.05) between each single-institution model and the big model. In the rectum, all averages of dosimetric parameters with the big model were lower than those with the single-institution models. An average difference of more than 10% was observed in V_50_ for the rectum for each case. For the PTV, there were similar SD values between the single-institution models and big model. However, for both the rectum and bladder V_50_, the big model had lower SD values compared with those for the single-institution model in each case.

**Table 3:** Mean ± SD values of dosimetric parameters and differences between big model versus single-institution model.

	Case I			Case II		
	Big model	Single-institution model	Difference (*p*-value)	Big model	Single-institution model	Difference (*p*-value)
**PTV**						
D_98_	71.2 ± 4.1	71.6 ± 4.2	− 0.3 ± 0.8 (0.588)	72.1 ± 3.5	72.2 ± 3.9	0.0 ± 0.8 (0.812)
D_95_	72.8 ± 4.2	72.9 ± 3.9	− 0.2 ± 0.6 (0.584)	74.3 ± 2.8	73.6 ± 3.4	0.7 ± 1.8 (0.789)
D_50_	79.0 ± 1.6	78.7 ± 0.5	0.3 ± 1.1 (0.416)	78.9 ± 1.5	78.8 ± 0.5	0.1 ± 1.2 (1.0)
D_2_	81.5 ± 1.8	81.6 ± 2.0	− 0.1 ± 1.5 (0.812)	81.2 ± 1.7	81.3 ± 1.6	0.0 ± 1.5 (0.786)
HI	0.13 ± 0.06	0.13 ± 0.07	0.00 ± 0.03 (1.0)	0.12 ± 0.05	0.12 ± 0.07	0.00 ± 0.03 (1.0)
**Rectum**						
V_90_	6.6 ± 4.0	8.4 ± 3.6	− 1.8 ± 1.6 (0.0625)	4.3 ± 2.7	6.0 ± 3.4	− 1.7 ± 1.9 (0.0625)
V_80_	12.0 ± 3.9	16.8 ± 1.5	− 4.8 ± 3.7 (0.0625)	9.3 ± 2.7	13.7 ± 1.8	− 4.3 ± 2.7 (0.0625)
V_50_	32.4 ± 3.8	46.6 ± 15.4	− 14.1 ± 13.8 (0.0625)	30.4 ± 3.9	42.5 ± 14.1	− 12.1 ± 15.2 (0.125)
**Bladder**						
V_90_	13.8 ± 1.8	13.3 ± 4.3	0.5 ± 3.3 (1.0)	7.2 ± 1.0	7.0 ± 2.3	0.2 ± 1.9 (1.0)
V_80_	19.4 ± 1.6	18.7 ± 4.1	0.7 ± 3.7 (1.0)	10.1 ± 0.8	10.0 ± 2.0	0.2 ± 1.8 (1.0)
V_50_	39.9 ± 2.0	38.4 ± 5.2	1.5 ± 5.6 (0.812)	21.5 ± 0.9	21.2 ± 2.6	0.3 ± 3.0 (0.855)

*D*_*98*_*, D*_*95*_*, D*_*50*_*, and D*_*2*_ Minimum dose in Gy to 2%, 50%, 95%, and 98% of the PTV; *HI* Homogeneity index; *V*_*90*_*, V*_*80*_*, and V*_*50*_ Volume ratio receiving 90%, 80%, and 50% of the prescribed dose.

**Figure 1:**
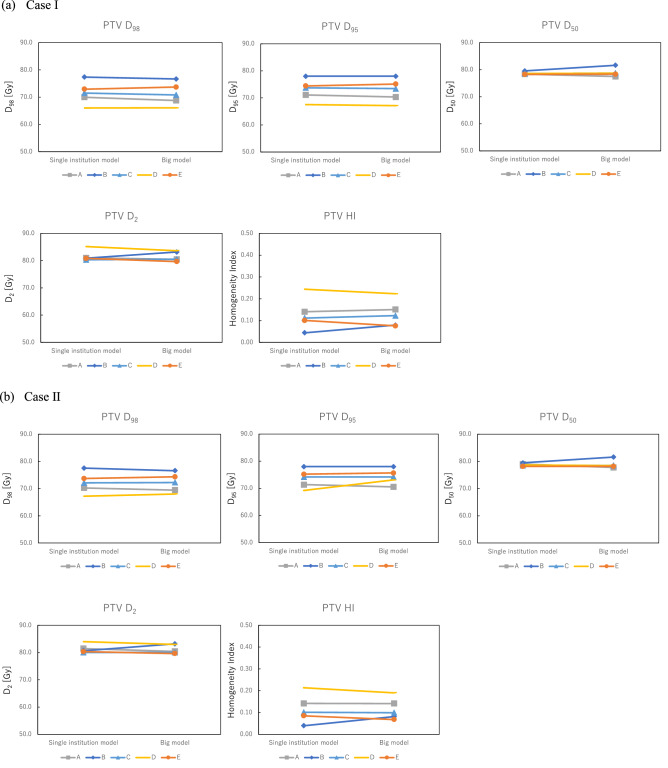

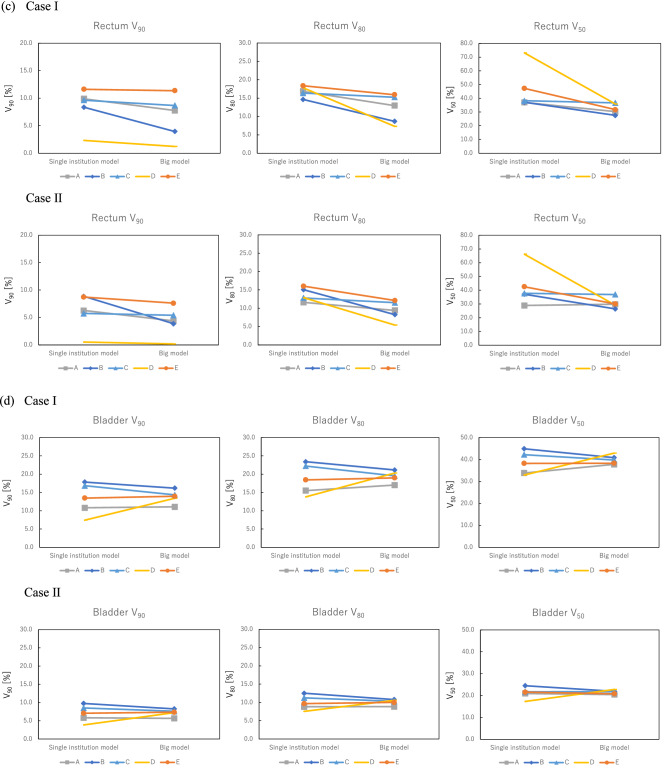
Dosimetric parameters for the (**a**, **b**) PTV, (**c**) rectum, and (**d**) bladder. For PTV, there were small differences between the single-institution models and big model in each case. Dosimetric parameters for the rectum calculated using the big model were lower than those calculated with the single-institution model. The volume ratio receiving 50% of the prescribed dose (V_50_) for institution D had the maximum difference (37.2%) between the big model and single-institution model. For the bladder, the dosimetric parameters calculated with the big model were lower than or equivalent to those calculated with the single-institution model, except for institution D.

Figure 1 shows the dosimetric parameter differences for the PTV, rectum, and bladder between the single-institution models and big model in each case. For the PTV, there were small differences between the single-institution models and big model. The maximum difference in D_95_ for the PTV among institutions was 3.9 Gy in institution D. Dosimetric parameters for the rectum calculated with the big model were lower than those calculated with the single-institution model. The maximum difference in V_50_ between the big model and single-institution model was 37.2% in institution D. The maximum differences among institutions for the single-institution model and big model were 9.3% and 10.2% for V_90_, 4.4% and 8.6% for V_80_, and 37.3% and 10.5% for V_50_, respectively. For V_50_, the big model was able to reduce the difference between each institution compared with each single-institution model. However, for both V_90_ and V_80_, the big model could not reduce the differences between each institution compared with each single-institution model. In the bladder, the dosimetric parameters calculated with the big model were lower than or equivalent to those calculated with the single-institution model, except for institution D. The maximum differences among institutions for the single-institution model and big model were 10.4% and 5.1% for V_90_, 9.6% and 4.1% for V_80_, and 12.0% and 5.1% for V_50_, respectively. In all dosimetric parameters, the big model had lower differences between institutions than the single-institution model.

### Model analytics

Table 4 shows R^2^ and χ^2^ values of regression models in each model. The R^2^ value calculated from regression lines between PCSs of DVH and GEDVH for the big model was comparable to those from each model. The χ^2^ value for the big model was the closest to 1.0 compared with each single-institution model. Table 5 shows the ratio and number of outliers for each index, such as CD > 4.0 ^17^, mZ > 3.5, SR > 3.0, and dA > 3.0^18^, for the rectum and bladder in the training data for each model. The ratio and number of outliers in the big model were comparable to those from each single-institution model.

**Table 4:** R^2^ and χ^2^ values for regression model in each model.

	R^2^		χ^2^	
	Rectum	Bladder	Rectum	Bladder
Big model	0.251	0.755	1.009	1.002
A	0.305	0.535	1.032	1.035
B	0.0084	0.86	1.119	1.031
C	0.436	0.809	1.145	1.077
D	0.652	0.893	1.04	1.058
E	0.0702	0.822	1.032	1.052

*R*^*2*^ Coefficient of determination; χ^*2*^ Chi squared.

**Table 5:** Ratio (%) and the number of outliers for each index.

Model (number)	CD > 4.0		mZ > 3.0		SR > 3.5		dA > 3.0	
	Rectum	Bladder	Rectum	Bladder	Rectum	Bladder	Rectum	Bladder
Big model (561)	0.5% 3	3.3% 19	4.8% 27	7.8% 44	0.4% 2	0.5% 3	0.5% 3	0.2% 1
A (123)	2.5% 3	5.8% 7	4,1% 5	9.1% 11	0.8% 1	0.0% 0	2.5% 3	0.0% 0
B (53)	5.7% 3	3.8% 2	11.3% 6	11.3% 6	0.0% 0	0.0% 0	5.7% 3	0.0% 0
C (20)	15.0% 3	10.0% 2	10.0% 2	10.0% 2	0.0% 0	0.0% 0	15.0% 3	0.0% 0
D (60)	5.0% 3	10.0% 6	11.7% 7	5.0% 3	0.0% 0	0.0% 0	5.0% 3	0.0% 0
E (100)	3.0% 3	8.0% 8	3.0% 3	6.0% 6	0.0% 0	0.0% 0	3.0% 3	6.0% 6

*CD* Cook’s distance; *mZ* Modified Z-score; *SR* Studentized residual; *dA* Areal difference of estimate.

The big model and single-institution A model covered all geometrical characteristics of cases I and II, while other single-institution models did not cover any geometric data for case I and II as follows:

institution B model: out-of-field volume percentage of the bladder; institution C model: bladder volume, overlap volume between target and OARs, and geometric distribution PCS of OARs; institution D model: out-of-field volume percentage of the bladder, overlap volume between target and the rectum, target volume, and geometric distribution PCS of the bladder; institution E model: target volume.

## Discussion

In this study, the multi-institution model (big model) was developed with 561 VMAT plans from five institutions with different planning strategies for prostate cancer. We evaluated the dose parameters of the VMAT plans generated with this big model. The big model could generate better or comparable dosimetric parameters compared with each single-institution model. The dosimetric parameters of the OARs were improved, especially V_50_, which can prevent radiation toxicity from occurring in the rectum and bladder during treatment^19^. Additionally, it can maintain coverage for the PTV and reduce inter-institution variation in the OARs.

The dose coverage of the PTV for the VMAT plan with the big model was comparable to the single-institution model, as shown in Table 3. It reflected the planning strategies of each institution, even though each institution used different prescribing methods. The original objective for the PTV at each institution in Table 2 could reflect the planning strategy of the VMAT plans with the big model. Thus, the big model could be used for several institutions by setting the PTV objectives for each institution’s planning strategy. Moreover, the VMAT plans with the big model could reduce the doses to the rectum at all institutions, as well as to the bladder at all institutions except for institution D, compared with the single-institution model in Fig. 1, although there were no significant differences. This is because the big model has a wide range of geometrical information from the 561 plans and thus could cover any geometrical characteristics of the patients. The geometric characteristics of cases I and II were out of the range in all single-institution models except for institution A. This indicates that the estimation accuracy of those models could potentially deteriorate, while the big model covered the anatomical characteristics of those cases. Tol et al. noted that the wide range of anatomical information in the RP model was important for generating better plan quality compared with the clinical plans^20^. The line objectives along the DVH lower bounds were also useful for optimizing the estimated DVHs predicted from the big model with the large number of combinations between anatomical and dosimetric characteristics of registered plans^15,21,22^. In the rectum, the big model could not reduce the differences in the V_90_ and V_80_ values between each institution compared with each single-institution model, as shown in Fig. 1. This is because the rectum V_90_ and V_80_ are areas that overlap with PTVs, and were affected by the different planning strategies of PTVs in each institution.

In model analysis, the big model regression line had an equivalent or superior goodness-of-fit compared with each single-institution regression line, as shown in Table 4. The ratios of outliers in the big model were also comparable to each single-institution in Table 5. These results indicate that the big model regression quality could be used in the same way as each single-institution model without the impact of outliers previously seen in other studies^11,23^.

The sharing of one RP model among multiple institutions can reduce the inter-institution variations from the reduction of SD values, as shown in Table 3, leading to standardization^14^. A previous study noted that an RP model is difficult to share among other institutions because of different planning strategies^12^. Our big model, as described in the current study, can cover any combination between anatomical and dosimetric characteristics based on the large number of plans, which can possibly overcome this issue. Therefore, sharing the big model generated from more plans found worldwide should realize the standardization of plan quality at any institution. For example, at a new institution, the planners will use the optimization parameters predefined by the big model, and then, they may customize those or use their own parameters in the case where those plans do not meet the dose criteria and/or planning strategy at that institution. The KBP can also serve as a training tool for the planners and institutions to implement the manual optimization^14^. One limitation is that this study included only two cases for evaluation, however: those were familiar prostate cancer cases; a study was performed to compare the dosimetric performance of the KBP models among five institutes^12^ and another one was used to evaluate whether the KBP models could improve dosimetric performance over the treatment period^14^. It is necessary to investigate more cases for various sites. A big model like the one presented here might also be applied to stereotactic radiotherapy because of its simple anatomical characteristics, while further study is needed for complicated anatomical cases such as head and neck cancer. The mechanical performance and delivery accuracy of the plans generated with the big model should also be verified before clinical use^24^.

## Conclusions

The big model, trained with over 500 clinical plans from multiple institutions with various planning strategies for targets and OARs in prostate cancer, could generate a superior or comparable plan quality compared with the VMAT plans generated with the single-institution models. Our work suggests a potential for plan quality standardization and reduction of inter-institution variability by using the big model.

## Data Availability

The dataset used and/or analyzed during the current study are available from the corresponding author on reasonable request.

## Abbreviations

IMRT: Intensity-modulated radiotherapy
VMAT: Volumetric modulated arc therapy
RP: RapidPlan
TPS: Treatment planning system
DVH: Dose volume histogram
OAR: Organ at risk
PTV: Planning target volume
GTV: Gross tumor volume
CTV: Clinical target volume
CT: Computed tomography
D_2_: Dose received by at least 2% of the volume
D_50_: Dose received by at least 50% of the volume
D_95_: Dose received by at least 95% of the volume
D_98_: Dose received by at least 98% of the volume
V_90_: Volume receiving 90% of the prescribed dose
V_80_: Volume receiving 80% of the prescribed dose
V_50_: Volume receiving 50% of the prescribed dose
HI: Homogeneity index
GEDVH: Geometrical dose volume histogram
PCS: Principal component score
R^2^: Coefficient of determination
χ^2^: Average chi squared
CD: Cook’s distance
mZ: Modified Z-score
SR: Studentized residual
dA: Areal difference of estimate
SD: Standard deviation
